# Corticosteroid therapy for primary treatment of Kawasaki disease – weight of evidence: a meta-analysis and systematic review of the literature

**Published:** 2009-08

**Authors:** Ganesh Athappan, Seth Gale, Thirumalaikolundusubramanian Ponniah

**Affiliations:** Caritas St Elizabeth Medical Centre, Tufts School of Medicine, Boston, USA; Caritas St Elizabeth Medical Centre, Tufts School of Medicine, Boston, USA; Caritas St Elizabeth Medical Centre, Tufts School of Medicine, Boston, USA

## Abstract

**Objective:**

Corticosteroids are the treatment of choice in most forms of vasculitis. However, their role in the primary treatment of Kawasaki disease (KD) is controversial. Our aim was to conduct a meta-analysis to assess the clinical course and coronary artery outcome of adding corticosteroids to standard therapy [intravenous immunoglobulin (IVIG) + aspirin] in patients with acute KD.

**Methods:**

We included randomised trials comparing the addition of corticosteroids to conventional primary therapy for Kawasaki disease.

**Results:**

A total of four studies were identified, which included 447 patients. The meta-analysis revealed a significant reduction in re-treatments with IVIG in patients receiving corticosteroid plus standard therapy compared with standard therapy alone [odds ratio (OR) 0.48; 95% confidence interval (CI): 0.24– 0.95]. There was however no significant reduction in the incidence of coronary artery aneurysms among patients who received corticosteroid therapy plus standard therapy, compared with standard therapy alone for either up to a month (OR 0.74; 95% CI: 0.23–2.40) or over one month ([OR 0.74; 95% CI: 0.37–1.51). Similarly no significant differences between treatment groups were noted in incidence of adverse events (OR 0.81; 95% CI: 0.05–0.88).

**Conclusion:**

The inclusion of corticosteroids in regimens for the initial treatment of Kawasaki disease decreased rates of re-treatment with intravenous immunoglobulin. However the addition of corticosteroids to standard therapy did not decrease the incidence of coronary aneurysms or adverse events.

## Summary

Kawasaki disease, first described in Japan in 1967, is now recognised as the leading cause of acquired heart disease in children in the developed world.^1^ It is a systemic vasculitis of unknown aetiology with a predilection for the coronary tree. Diagnosis of KD^2^ requires the presence of fever lasting five days or more, combined with at least four of the following five physical findings, without an alternative explanation: (1) bilateral bulbar conjunctival injections, (2) oral mucous membrane changes which include injected or fissured lips, injected pharynx, or strawberry tongue, (3) peripheral extremity changes, including erythema of palms or soles, oedema of hands or feet, and periungual desquamation, (4) polymorphous rash, and (5) cervical lymphadenopathy with at least one lymph node > 1.5 cm in diameter. Children who do not meet the criteria may have an incomplete or atypical form of KD. Although cardiac involvement is not recognised in the diagnostic criteria, coronary artery aneurysms are the most common cause of morbidity and mortality in patients with KD.^3^

The pathology of acute KD reveals a panvasculitis of the small and medium-sized muscular arteries with endothelial oedema, necrosis, and leukocyte infiltration of the arterial wall.^4^ Blood vessel damage in KD is produced by immune-mediated injury to the arterial wall. Accordingly, corticosteroids with their inherent anti-inflammatory properties would be expected to complement high-dose IVIG and aspirin in the treatment of acute KD. However, available reports on the use of steroids in this setting are contradictory.

In the pre-IVIG era, early studies observed a high rate of coronary aneurysms in children treated with corticosteroids as primary therapy for KD.^5^ A few subsequent studies however showed either no ill effects or possible improved outcomes with the use of steroids.^6^-^10^ Two recent, randomised, controlled studies have added some conflicting data to the controversy surrounding the use of corticosteroids for the primary treatment of KD.^11^,^12^ We therefore performed a meta-analysis to ascertain whether the addition of corticosteroids to IVIG for the primary treatment of KD might improve clinical outcomes.

## Methods

We performed a computerised search of Ovid, Google Scholar and PubMed databases from January 1975 to June 2008, and reviewed cited references to identify the relevant studies. Citations were screened at the title/abstract level and retrieved as full reports. Search keywords were ‘Kawasaki’ and ‘corticosteroids’ in single use and in combination. Studies were included if the following criteria applied: (1) English language articles (2) randomised, controlled trials comparing standard therapy (IVIG and aspirin) with the addition of steroids to standard therapy, (3) involved patients with primary Kawasaki disease within 10 days of fever and (4) two-dimensional echocardiography or coronary artery catheterisation was performed at least one week after therapy to detect the presence of coronary aneurysms.

Exclusion criteria were as follows: (1) non-randomised studies, (2) included patients for rescue therapy – failed initial standard therapy, (3) duplicate publication, (4) ongoing/unpublished study, and (5) publication only as an abstract or as conference proceedings. The primary endpoint was coronary artery abnormality (CAA) by echocardiographic assessment within and after a month. Coronary arteries were classified on the basis of the presence or absence of aneurysms according to criteria of the Japanese Ministry of Health in all included studies. Secondary endpoints included adverse events and re-treatment – initial treatment failure requiring additional therapy.

Data were extracted from each study into a structured spreadsheet and absolute numbers were recalculated where percentages were reported. The present meta-analysis was carried out following the guidelines of the *Cochrane Handbook of Systematic Reviews of Interventions version* 5.0.0.^13^

## Statistical analysis

Data analysis was performed using Review Manager software version 5.^14^ Continuous variables were entered as means, and categorical variables as *n*%. A random-effects model was used for pooled analysis, and 95% confidence intervals (95% CI) were used to establish the precision of our results. If the CI did not cross one, the results were considered to be statistically significant. Statistical heterogeneity of the study results was examined using Q statistic and the I2 test. A rigorous analysis of all studies was done to look for methodological, clinical or statistical heterogeneity. Significant heterogeneity was considered present for *p* values < 0.10 and/or an I2 ≥ 50%. Data are presented as odds ratios (OR) with 95% CIs and statistical significance set at *p* < 0.05 (two-tailed).

## Results

Our search identified six randomised, controlled trials on Kawasaki disease with steroid therapy as add-on to standard therapy.^11^,^12^,^15^-^18^ Two of these articles were excluded as they did not meet the inclusion criteria. The article by Higashi *et al.* studied impairment of angiogenic activity in the serum of patients with Kawasaki disease.^15^ The other trial by Hashino *et al.* assessed the role of steroids in patients with re-treatment for immunoglobulin-resistant Kawasaki disease.^16^

The four studies that met the inclusion criteria included 447 patients. Of these, 223 were treated with corticosteroids in addition to standard therapy, while the remaining 224 received only standard therapy. The characteristics of the analysed trials are presented in Table 1.

**Table 1:** Characteristics Of Included Studies

	*Patient characteristics*											
								*IVIG*		*Corticosteroids (starting regimen)*		
*Study name*	*Total (n)*	*Standard therapy*	*Standard therapy + corticosteroids*	*Male (%)*	*Age (years)*	*Maximum days before enrollment*	*Aspirin regimen (mg/kg)*	*Dose (mg/kg)*	*Number of doses*	*Preparation*	*Dosage (mg/kg)*	*Frequency (times/day)*
Inoue 2006	178	88	90	57.3	4.5	9	30	1	2	pednisolone	2	3
Newburger 2007	199	98	101	62	2.9	10	80–100	2	1	IV methylprednisolone	30	1
Okada 2003	32	18	14	56.25	2.75	9	30	1	2	pednisolone	2	3
Sundel 2003	39	21	18	69	-	10	20–25	2	1	IV methylprednisolone	30	1

**Fig. 1.**
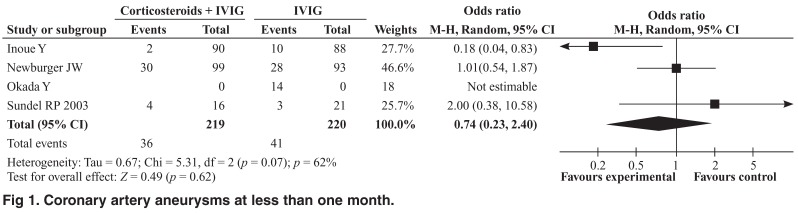
Coronary artery aneurysms at less than one month.

**Fig. 2.**
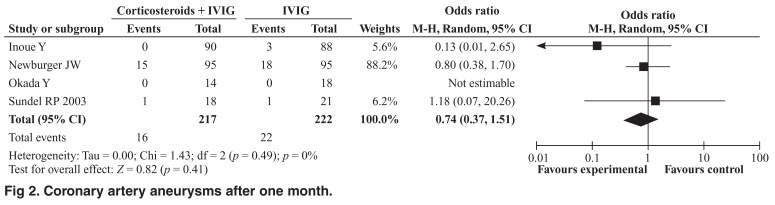
Coronary artery aneurysms after one month.

**Fig. 3.**
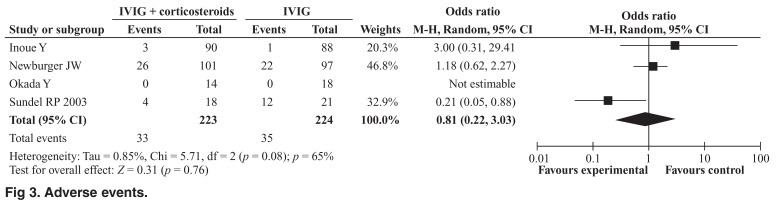
Adverse events.

The meta-analysis revealed no significant reduction in the incidence of coronary artery aneurysms among patients who received corticosteroid therapy plus IVIG compared with IVIG alone for up to a month (OR 0.74; 95% CI: 0.23– 2.40, Cochrane Q = 5.31, I2 = 62%) (Fig. 1) or for more than a month (OR 0.74; 95% CI: 0.37–1.51, Cochrane Q = 1.43, *I*^2^ = 0) (Fig. 2). However, when considered individually, the study by Inoue *et al*. showed a significant reduction in the incidence of coronary aneurysms up to a month (2.2 % vs 11.4, *p* = 0.017). The incidence of adverse events was similar in the two treatment groups (OR 0.81; 95% CI: 0.22–3.03, Cochrane Q = 5.71, *I*^2^ = 65%) (Fig. 3).

**Fig. 4.**
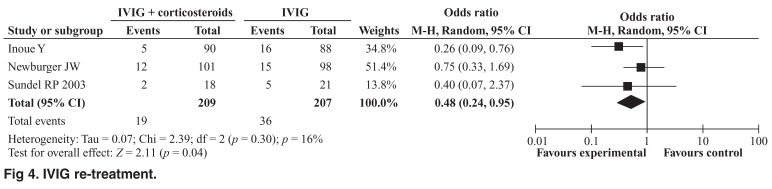
IVIG re-treatment.

Repeat IVIG therapy was significantly higher in patients undergoing standard therapy alone compared to those treated with a combination of standard therapy and steroids. Pooled analysis of this endpoint demonstrated better outcomes with incorporation of steroids in the primary treatment regimen (OR 0.48; 95% CI: 0.24–0.95, Cochrane Q = 2.39, *I*^2^ = 16%) (Fig. 4).

## Discussion

Kawasaki disease is the primary cause of acquired heart disease in children in the western world.^1^ It is typically an acute, self-limiting, multi-system vasculitis of childhood. Nevertheless, if left untreated, cardiovascular complications, particularly coronary artery (CA) aneurysms may develop and lead to significant morbidity and mortality. Therefore, the current standard of care for KD includes expeditious diagnosis and timely treatment with high-dose IVIG and aspirin for rapid resolution of the inflammation and prevention of coronary artery abnormalities. Nevertheless, approximately 3 to 5% of children on the present standard of care develop coronary aneurysms.^19^ To improve patient care, several researchers have considered the addition of corticosteroids to the present standard of care, based on their anti-inflammatory properties. However, existing data on their role in the primary treatment of KD is unclear.

Our meta-analysis of the existing data did not support their administration in addition to standard therapy for the primary prevention of coronary anomalies in patients with KD. The results of our analysis do not correlate with observations made in several previous studies, including those of Inoue *et al.* or those of a previous meta-analysis on the same topic.^11^,^12^ The differing outcomes of incidence of coronary aneurysms may be explained by the IVIG regimens used in the individual studies. Newburger *et al.* and Sundel *et al.* used a regimen of single-dose 2 g/kg, whereas Inoue and other Japanese trials have used a regimen of 1 g/kg on two consecutive days. The benefits of corticosteroids may therefore have been eclipsed by administration of a more effective IVIG regimen. The American Heart Association and the AAP guidelines recommend that IVIG be administered as a single dose of 2 g/kg for children, based on compelling evidence that single infusion of IVIG (2 g/kg) is superior to any split regimen.^20^

While corticosteroid use in primary KD treatment to prevent CAA seems untenable at the moment, primary corticosteroid use does appear to significantly reduce the need for re-treatment with IVIG. Therefore, it appears that there might be a subset of patients at risk of failing standard therapy who might benefit from initial glucocorticoid therapy. Additional risk stratification data at presentation are required to allow selection of patients who might benefit from early corticosteroid administration.

Limitations of this study need to be acknowledged. Disparate study designs and very few patients pooled in the meta-analysis resulted in statistical heterogeneity such that the meaningfulness of any overall estimate could be called into question. The conclusions of our meta-analysis may therefore be misleading and cannot support or preclude the usage of initial glucocorticoid regimens in the primary treatment of KD.

## Conclusion

The inclusion of corticosteroids in regimens for the initial treatment of Kawasaki disease decreases rates of treatment failure but does not significantly reduce the incidence of coronary aneurysms or adverse events. Future randomised, controlled studies should focus on risk stratification of KD patients and identifying those who might benefit from primary therapy with corticosteroids.
